# Orchestration of Dopamine Neuron Population Activity in the Ventral Tegmental Area by Caffeine: Comparison With Amphetamine

**DOI:** 10.1093/ijnp/pyab049

**Published:** 2021-07-19

**Authors:** Ornella Valenti, Alice Zambon, Stefan Boehm

**Affiliations:** Division of Neurophysiology and Neuropharmacology, Center for Physiology and Pharmacology, Medical University Vienna, Vienna, Austria

**Keywords:** Adenosine receptor antagonists, amphetamine, caffeine, psychostimulants, VTA dopamine neurons

## Abstract

**Background:**

Among psychostimulants, the dopamine transporter ligands amphetamine and cocaine display the highest addictive potential; the adenosine receptor antagonist caffeine is most widely consumed but less addictive. Psychostimulant actions of amphetamine were correlated with its ability to orchestrate ventral tegmental dopamine neuron activity with contrasting shifts in firing after single vs repeated administration. Whether caffeine might impinge on dopamine neuron activity has remained elusive.

**Methods:**

Population activity of ventral tegmental area dopamine neurons was determined by single-unit extracellular recordings and set in relation to mouse behavior in locomotion and conditioned place preference experiments, respectively.

**Results:**

A single dose of caffeine reduced population activity as did amphetamine and the selective adenosine A2A antagonist KW-6002, but not the A1 antagonist DPCPX. Repeated administration of KW-6002 or amphetamine led to drug-conditioned place preference and to unaltered or even enhanced population activity. Recurrent injection of caffeine or DPCPX, in contrast, failed to cause conditioned place preference and persistently reduced population activity. Subsequent to repetitive drug administration, re-exposure to amphetamine or KW-6002, but not to caffeine or DPCPX, was able to reduce population activity.

**Conclusions:**

Behavioral sensitization to amphetamine is attributed to persistent activation of ventral tegmental area dopamine neurons via the ventral hippocampus. Accordingly, a switch from acute A2A receptor-mediated reduction of dopamine neuron population activity to enduring A1 receptor-mediated suppression is correlated with tolerance rather than sensitization in response to repeated caffeine intake.

**Significance Statement**

This work reveals that a single dose of caffeine suppresses population activity of dopamine neurons in the ventral tegmental area (VTA) via A2A receptors in a manner similar to that of amphetamine. In a drug-conditioned place preference paradigm, amphetamine and A2A receptor antagonism led to drug-related place preference, whereas caffeine and A1 receptor antagonism failed to do so. VTA dopamine neuron population activity was reduced after repeated caffeine or A1 antagonist administration but unaltered or even enhanced after repetitive A2A antagonist and amphetamine application, respectively. As persistent activation of VTA dopamine neuron activity is correlated with behavioral sensitization by amphetamine, the switch from A2A receptor-mediated reduction of dopamine neuron population activity to enduring A1 receptor-mediated suppression, as described here, can be viewed as correlate of tolerance rather than sensitization in response to repeated caffeine intake.

## Introduction

Psychostimulants cause hyperlocomotion, arousal, vigilance, attention, and anorexia (Wood et al., 2014). Among representatives as diverse as amphetamine, cocaine, methylphenidate, and caffeine, the latter is most widely consumed as it is contained in plants and ingested through beverages made therefrom (Heckman et al., 2010). In addition to psychomotor activation, psychostimulants have reinforcing properties, and both effects rely on increased dopaminergic neurotransmission (Fredholm et al., 1999; Ferré, 2016). Mechanistically, these drugs either bind to dopamine (DA) transporters and inhibit reuptake and/or promote reverse transport (Iversen, 2006) or act as antagonist at adenosine A1 and A2A receptors, as does caffeine (Fredholm et al., 1999; Ferré, 2016). However, these receptors form heteromers with DA, D1 and D2 receptors, respectively, and thereby caffeine may impinge on dopaminergic signaling (Fuxe et al., 2010). Additionally, blockade of presynaptic adenosine receptors affects DA release (Ferré, 2008, 2010).

The DA motive system that is key for psychostimulant actions comprises 2 main sections: DA neurons in the substantia nigra projecting to the dorsal striatum are involved in action selection and motor behavior, whereas DA neurons in the ventral tegmental area (VTA) projecting to the nucleus accumbens (NAcc) are associated with motivation and reinforcement learning (Volkow et al., 2017). Even though the segregation between these 2 pathways is less pronounced than assumed originally (Wise, 2009), their roles in the actions of psychostimulants appear to be separate. For caffeine, signal integration via A2A-D2 receptor heteromers in striatal neurons is viewed as a key element in its psychostimulant activity (Ferré, 2016), while plasticity in the firing pattern of VTA DA neurons appears decisive for amphetamine actions (Grace, 2010a).

DA neurons in the VTA fire either in continuous tonic or burst-like phasic modes (Grace and Bunney, 1984a, 1984b; Grace et al., 2007). The latter is triggered by salient stimuli such as reinforcing or aversive cues. Tonic firing causes low-level DA release in NAcc, leads to preferential activation of high-affinity D2 receptors, and determines motivational arousal. Phasic firing, in contrast, results in extracellular DA sufficiently high to stimulate low-affinity D1 receptors in NAcc, and this is assumed to underlie conditioning to positive or negative reinforcers (Grace, 2010b; Volkow et al., 2017). The firing pattern of VTA DA neurons is governed by projections from other brain regions. The switch from tonic to phasic firing, for instance, is contingent on glutamatergic drive from the pedunculopontine tegmentum. As this is mediated by NMDA receptors, the shift into bursting can only occur at depolarized membrane potentials as to be found in spontaneously active neurons. Therefore, the more neurons display tonic firing, the more can go into burst firing (Lodge and Grace, 2006). The proportion of DA neurons with spontaneous action potentials is known as “population activity” (Grace, 2000). The latter is restricted by GABAergic input from the ventral pallidum (VP), which, in turn, is under control of the ventral hippocampus (vHPC) via NAcc (Floresco et al., 2003).

Acute injection of either amphetamine (Lodge and Grace, 2008) or cocaine (Lodge and Grace, 2005) reduces VTA DA neuron population activity, whereas repeated amphetamine administration followed by withdrawal leads to respective increases (Lodge and Grace, 2008). These changes in VTA population activity were paralleled by altered locomotor behavior (Lodge and Grace, 2008). For caffeine, an inhibitory effect on spontaneous action potential frequencies in VTA DA neurons has been described (Stoner et al., 1988), but it remained unknown whether this psychostimulant might affect population activity in a manner similar to that of amphetamine and cocaine. Accordingly, changes in VTA DA neuron population activity caused by caffeine and selective adenosine receptor ligands were investigated and compared with those of amphetamine. The results reveal conspicuous differences between adenosine antagonists and amphetamine.

## Materials and Methods

### Materials

Isoflurane (Forane) was from AbbVie (Vienna, Austria); Chicago Sky Blue from Alfa Aesar (Karlsruhe, Germany); and KW-6002 (istradefylline), DPCPX (8-Cyclopentyl-1,3-dipropylxanthine), and CGS21680 (4-[2-[[6-Amino-9-(N-ethyl-β-D-ribofuranuronamidosyl)-9H-purin-2-yl]amino]ethyl]benzene-propanoic acid hydrochloride) from Tocris (Abingdon, UK). D-Amphetamine sulfate, caffeine, chloral hydrate, and bulk chemicals were from Sigma-Aldrich (Vienna, Austria). Amphetamine and caffeine were dissolved in 0.9% saline, and KW-6002 and DPCPX were dissolved in dimethyl sulfoxide as vehicle (1:30 in saline).

### Animals

A total of 146 adult (2–4 months of age) C57BL/6J male mice were used in this study. Mice were bred in our facilities and housed at 22°C and a humidity of 47% with 12-hou-light/-dark cycles (lights on at 7 am) and ad libitum access to food and water. Experiments were performed in accordance with the current European Directive for the use of animals for scientific purposes and with regulations of the Medical University of Vienna and ARRIVE guidelines under an animal experimentation license approved by the Austrian Ministry of Science (GZ 66.009/0382-WF/V/3b/2017).

### Conditioned Place Preference (CPP) and Locomotor Activity

CPP training was conducted according to established protocols (Cunningham et al., 2006; Steinkellner et al., 2014) in a commercial apparatus that consists of 4 aligned boxes (MED Associates; product # MED-OFAS-MSU) plus 4 inserts (MED Associates; product # ENV-512); each insert is made of 2 chambers with distinguishable floor (grid vs rod). An unbiased subject assignment procedure was employed (Cunningham et al., 2006), and drug treatments were assigned randomly: saline (0.9% NaCl; N = 14), amphetamine (1.5 mg/kg; n = 14), caffeine (5 mg/kg; n = 13), KW-6002 (1.5 mg/kg; n = 14), or DPCPX (4 mg/kg; n = 12). The very same dose of amphetamine has been reported to affect VTA DA neuron population activity (Lodge and Grace, 2008); this dosage of caffeine was chosen as it is known to promote locomotion as well as CPP (Fredholm et al, 1999). DPCPX and KW-6002 were used at doses that had been employed before to selectively block A1 and A2A receptors, respectively (e.g., El Yacoubi et al., 2000; Gołembiowska et al., 2013).

Mice were single-housed and habituated to handling and transportation for 5 to 7 days; all manipulations took place during the light cycle and were started at 8:00 am. Experiments comprised preconditioning (day 0), conditioning (days 1 to 6), and test phases (day 7). Each session lasted 30 minutes. During preconditioning (pre-test), mice were transferred to 1 of the boxes and allowed to explore both chambers. On day 1, mice were injected with either the pre-assigned drug or saline and placed in the pre-assigned cue-specific chamber; access to the neighboring chamber was prevented by a barrier. Data recorded on day 1 were used for the analysis of drug-induced locomotor activity (Figure 2A). On day 2, mice received either drug-vehicle (dimethyl sulfoxide 1:30 mL in saline) or saline and were positioned in the other respective chamber. The protocol of days 1 and 2 was repeated on days 3 to 6. Thus, for mice assigned to drug treatment (amphetamine, caffeine, KW-6002, or DPCPX), 3 drug injections were alternated with 3 vehicle injections, whereas the control group received saline from day 1 to day 6. On test day (day 7), none of the mice received any injections, the barrier was removed, and drug-free mice were allowed to explore both compartments; place preference was determined by calculating the time spent in each of the 2 chambers. All sessions were recorded using the Active Monitor Software of the MED-PC IV software package (MedAssociates) and analyzed offline. Distances covered by the mice were determined by beam-breaks.

### Surgery, In Vivo Single-Unit Recordings, and Drug Administration

Single-unit extracellular recordings were conducted as previously described, with minor modifications (Valenti and Grace, 2010). Mice assigned to the acute drug experimental protocols were transferred to the recording room and anesthetized on the same day of the experiment. CPP mice were anesthetized within 30 minutes from the CPP test (day 7) and randomly assigned to 1 of the protocols described below. Anesthesia was induced with 3% isoflurane, followed by 8% chloral hydrate (400 mg/kg; i.p.), which was also used for maintenance (280 mg/kg; i.p.). Subjects were secured on a stereotactic device (David Kopf Instruments), heart rate was monitored, and core temperature was regulated by a heating pad (Harvard Apparatus). Coordinates of brain regions were calculated using the Mouse Brain Atlas (Franklin and Paxinos, 2008). Extracellular recordings were conducted from the VTA (from Bregma, in mm P: 3; L: 0.4). In some experiments, cannulas were implanted within VP (P: 0; L: 1.6; V: 3.8) or NAcc (A: 1.6; L: 0.7; V: 3.5).

**Figure 1.**
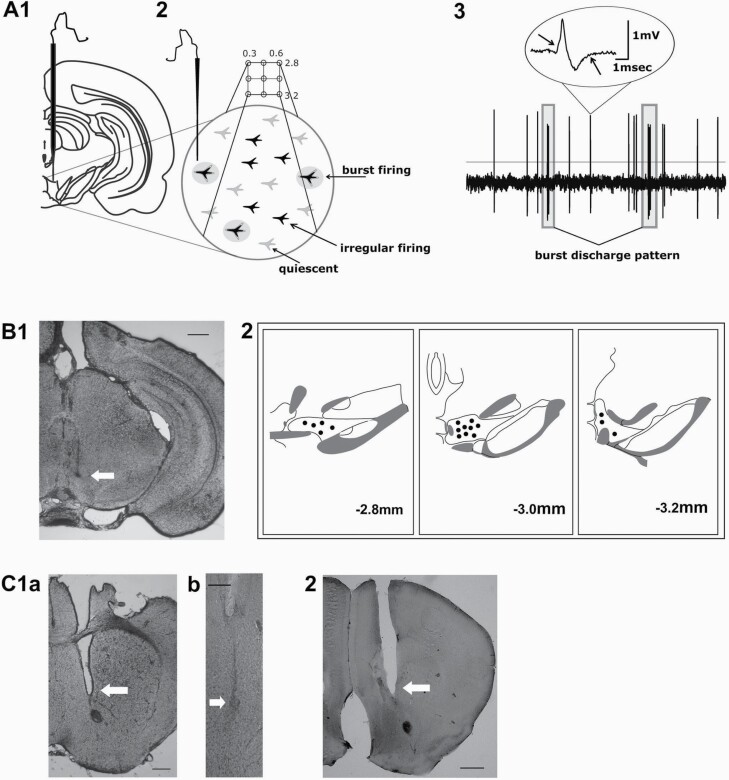
Single-unit extracellular recordings from ventral tegmental area dopamine neurons and post-hoc histological analysis. (A) Schematic illustration of single-unit recordings of VTA DA neuron population activity. (1) Glass microelectrodes were lowered to reach the upper edge of VTA and then slowly advanced while scanning for spontaneously active neurons; a dorsal to ventral electrode trajectory through the VTA is termed track (from surface, −4 to −5mm). (2) Top: after completion of the first trajectory, VTA measurements continued for another 5 to 8 tracks in a predetermined pattern. Bottom: activity pattern of VTA DA neurons. Approximately 50% of all VTA neurons are quiescent (light-grey) under baseline conditions; when active, DA neurons exhibit either irregular firing (black) or burst firing (black surrounded by grey circles). (3) Original trace of a representative recording with 2 burst firing events being indicated. The inset shows an enlarged action potential waveform typical of a DA neuron that is characterized by a notch and a prominent after-hyperpolarization, both indicated by arrows. (B) (1) Shows a coronal section from a mouse brain with the position of the recording electrode marked by iontophoretically injected Chicago Sky Blue (indicated by the arrow); (2) schematic drawings with spots indicating electrode positions in representative experiments; numeric values at the bottom indicate posterior distances of the section from bregma in mm. (C) Examples of cannula placements for drug application into ventral pallidum (1a) and nucleus accumbens (2), respectively; for VP, a cannula position is also shown at higher magnification (1b). Scale bars reflect 500 µm (C1a and 2) and 100 µm (1b), respectively.

Pipettes were fabricated from borosilicate glass capillaries (1.2-mm-outer-diameter filament glass; Harvard Apparatus) with a vertical puller (PE-22, Narashige) and filled with 2% Chicago Sky Blue in 0.5M NaCl (in situ resistance 10–15 MΩ). Electrodes were lowered into the region of interest using a micromanipulator (S-IVM-1000, Scientifica) and slowly advanced until spontaneously firing cells were encountered (Figure 1A1–2). Neurons were isolated, and baseline activity was recorded for up to 5 minutes (Figure 1A3). Following drug administration, electrodes were advanced further to reach the end of the first track (from brain surface; VTA: 4–5 mm). Thereafter, recordings continued for another 5 to 8 vertical tracks in a predetermined pattern across horizontal and vertical axes (Figure 1A2). Signals were amplified by a headstage pre-amplifier, fed into an ELC-03XS universal amplifier (1000× gain, 100- to 4000-Hz bandpass; NPI Electronics), and digitized at 20 kHz (Power1401 A/D board; CED).

For systemic effects, agents were injected i.p.; we employed vehicle and the very same drugs as used during CPP training. To identify sites of actions, drugs or tetrodotoxin were applied to specific brain areas using metallic cannulas (Plastics One; 31 GA). The latter were left in place for at least 10 minutes after infusion. Recordings started 30 minutes after drug administration.

Open filter settings (low pass, 30 Hz; high pass, 16 kHz; DPA-2F filter, NPI Electronics) were used to allow for proper post-hoc classification of neurons. At the end of experiments, mice were killed with a lethal dose of anesthetic; spots of electrodes were marked via electrophoretic ejection of Chicago Sky Blue (Fintronics Inc., USA). Electrode and cannula positions were confirmed by post-hoc histology (Figure 1B–C).

### DA Neuron Classification, Data Analysis, and Statistics

DA neurons were classified by well-established criteria (Valenti and Grace, 2010; Ungless and Grace, 2012). They exhibited biphasic action potentials with broad waveforms (>2.2 ms), a “notch” in the rising phase, and pronounced afterhyperpolarizations (Figure 1A3, insert). Firing occurred in 2 modalities: irregular or burst firing (Grace and Bunney, 1984a, 1984b; Valenti and Grace, 2010). Analysis was performed with built-in functions in Spike2 (version 7.01; CED) (Forro et al., 2013). Spike waveforms were sorted into clusters of putative units and assigned to individual neurons on the basis of their vector clouds and manual inspection. Three parameters were determined: (1) population activity as number of spontaneously active neurons per electrode track; (2) average firing rate; and (3) percentage of action potentials occurring in burst discharges; such bursts comprise events with >2 spikes that start with inter-spike intervals of <80 ms and end when inter-spike interval exceeds 160 ms.

Statistical differences were assessed using GraphPad or MATLAB and regarded as significant at *P* < .05; for clarity, we have only reported variations compared with saline or caffeine. For all experimental protocols, results obtained with saline injection were neither significantly different from each other nor from results obtained after vehicle injection (1-way ANOVA followed by Bonferroni Multiple Comparison test). For none of the experimental protocols did administration of drugs elicit significant changes in firing rates or percent burst firing compared with saline (1-way ANOVA followed by Bonferroni multiple comparison test).

For the analysis of rate distribution, interpolation curves were designed by using built-in functions of Microsoft Excel. Briefly, from the numerical values obtained in our recordings, several polynomial equations were generated, and the 1 line fit that described our data best was selected; the chosen polynomial equations are indicated in figure legends.

All data are reported as arithmetic means ± SEM; N indicates numbers of mice, n gives numbers of neurons. Differences between multiple groups were analyzed by 1-way or 2-way ANOVA followed by Bonferroni multiple comparison.

## Results

### Single Doses of Caffeine Affect VTA DA Neuron Population Activity by Blockade of A2A Adenosine Receptors in the Same Way as Amphetamine

**Figure 2.**
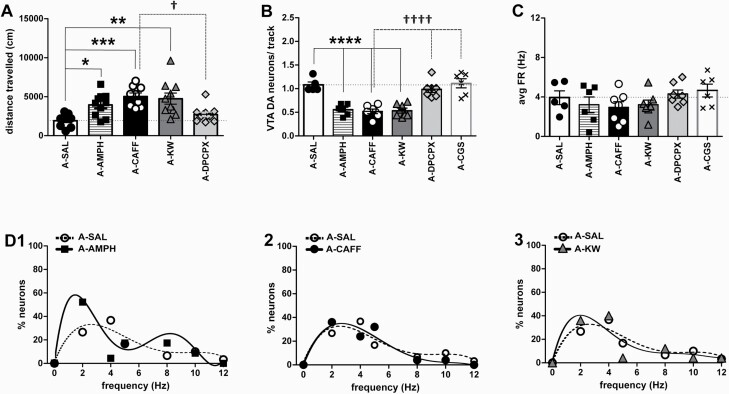
Effects of acute administration of caffeine and selective adenosine receptor ligands on locomotion and dopamine neuron population activity: comparison with amphetamine. (A) Locomotor activity (distance travelled in cm) in response to systemic administration of amphetamine (AMPH; 1.5 mg/kg; N = 10), caffeine (CAFF; 5 mg/kg; N = 9), KW-6002 (KW; 1.5 mg/kg; N = 10), DPCPX (4 mg/kg; N = 11), or saline (SAL; N = 9). *P < .05, **P < .01, and ***P < .001 vs saline. †*P* < .05 vs caffeine (dashed line) (1-way ANOVA followed Bonferroni Multiple Comparison test; F_(4,44)_ = 7.69; *P* < .0001). (B) DA neuron population activity (neurons/track) in response to systemic administration of amphetamine (n = 27, N = 6), caffeine (n = 28, N = 7), KW-6002 (n = 27, N = 7), DPCPX (n = 45, N = 7), CGS-21680 (CGS; 0.1mg/kg; n = 54, N = 6), and saline (n = 39, N = 5), respectively. *****P < .0001 vs saline; ††††*P* < .001 vs caffeine (dashed line) (1-way ANOVA followed by Bonferroni Multiple Comparison test; F_(5,32)_ = 20.56; *P* < .0001). (C) Average firing rates (avg FR) of DA neurons in response to systemic drug administration as in B; *P* = .265 (1-way ANOVA followed by Bonferroni Multiple Comparison test; *P* = .2652). (D) Firing rate distribution in response to acute administration of amphetamine (1), caffeine (2), and KW-6002 (3), respectively. For comparison, the firing rate distribution after saline injection is included in each of these graphs. Polynomial equations, A-SAL: y = −0.0245x^4^ + 0.7607x^3^ − 8.0082x^2^ + 29.262x − 1.0606; A-AMPH: y = −0.0084x^6^ + 0.3334x^5^ − 5.097x^4^ + 37.217x^3^ − 128.93x^2^ + 170.8x − 2E-08; A-CAFF: y = −0.0142x^4^ + 0.5215x^3^ − 6.4053x^2^ + 26.263x + 0.8312; A-KW: y = −0.0455x^4^ + 1.3131x^3^ − 12.53x^2^ + 40.167x − 0.5974.

A prototypic effect of psychostimulants is hyperlocomotion (Wood et al., 2014). Therefore, drugs were tested for locomotor responses first. The i.p. administration of 5 mg/kg caffeine or 1.5 mg/kg of A2A receptor antagonist KW-6002 increased distances travelled by mice by a factor of 2 compared with saline. A similar effect was observed with 1.5 mg/kg amphetamine, but not with 4 mg/kg DPCPX (Figure 2A; 1-way ANOVA followed by Bonferroni Multiple Comparison Test; F_(4,44)_ = 7.69; *P* < .0001).

Locomotor effects of amphetamine have been correlated with changes in population activity of VTA DA neurons (Grace, 2010b). To explore whether actions of adenosine receptor ligands on locomotion might be related to changes in the activity of such neurons, we conducted in vivo single-unit recordings (Figure 1A–B). Under control conditions, an average of 1.1 ± 0.08 spontaneously active VTA DA neurons were encountered per electrode track; neurons exhibited average firing rates of 3.9 ± 0.7 Hz with 32.5 ± 5.3% of action potentials occurring in bursts (n = 39, N = 5). Amphetamine decreased population activity (Figure 2B), as reported previously (Lodge and Grace, 2008), and an equivalent effect was seen with caffeine and KW-6002. For these 3 agents, values of population activity did not differ from each other. Vice versa, population activity values were the same for saline, the A1 receptor antagonist DPCPX, and the A2A agonist CGS21680 (Figure 2B; 1-way ANOVA followed by Bonferroni Multiple Comparison Test; F_(5,32)_ = 20.56; *P* < .0001). None of these drugs affected either average firing rates (Figure 2C; 1-way ANOVA followed by Bonferroni Multiple Comparison Test, *P* = .2652) or the percentage of neurons firing in bursts (data not shown; 1-way ANOVA followed by Bonferroni Multiple Comparison Test, *P* = .5218), but amphetamine altered the firing rate distribution (Figure 2D1) as described before (Lodge and Grace, 2008); this effect was not shared by any of the adenosine antagonists (Figure 2D2–D3) neither by the A2A agonist CGS21680 (data not shown). These results indicate that caffeine is capable of orchestrating population activity of VTA DA neurons via A2A receptors in a manner similar to, but not identical with, that of amphetamine. Given that none of the drug treatments produced significant changes in firing rates and burst activity, these data have been omitted from the following sections.

### Caffeine and Amphetamine Exploit Different Neuronal Circuits to Control VTA DA Neurons

**Figure 3.**
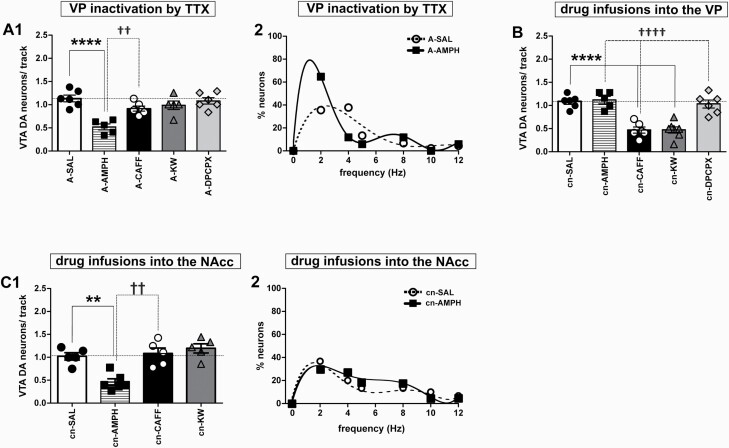
Roles of nucleus accumbens and ventral pallidum in the acute effects of caffeine, selective adenosine receptor ligands, and amphetamine on DA neuron population activity. (A) (1) DA neuron population activity (neurons/track) in response to systemic administration of amphetamine (AMPH; n = 19, N = 7), caffeine (CAFF; n = 44, N = 6), KW-6002 (KW; n = 36, N = 5), DPCPX (n = 49, N = 6), and saline (SAL; n = 54, N = 6), respectively. Experiments were executed after inactivation of VP by injection of tetrodotoxin (TTX) (1 µM). ***P < .001 vs saline; ††*P* < .01 vs caffeine (dashed line) (1-way ANOVA followed by Bonferroni Multiple Comparison test; F_(4,27)_ = 11.06; *P* < .0001). (2) Firing rate distribution in response to systemic administration of either amphetamine or saline (as in A1) subsequent to inactivation of VP by TTX. Polynomial equations, A-SAL: y = −0.0023x^5^ + 0.0395x^4^ + 0.2336x^3^ − 7.0686x^2^ + 31.375x − 0.1924; A-AMPH: y = −0.0032x^6^ + 0.1494x^5^ − 2.6782x^4^ + 22.932x^3^ − 93.648x^2^ + 147.06x − 3E-08. (B) DA neuron population activity (neurons/track) in response to intrapallidal cannula infusion of amphetamine (cn-AMPH; 10 µM; n = 41, N = 5), caffeine (cn-CAFF; 100 µM; n = 25, N = 6), KW-6002 (cn-KW; 10 µM; n = 26, N = 7), DPCPX (cn- DPCPX; 1 µM; n = 47, N = 6), and saline (cn-SAL; n = 54, N = 6), respectively. ***P < .001 vs saline; †††*P* < .001 vs caffeine (dashed line) (1-way ANOVA followed by Tukey’s Multiple Comparison test; F_(4,25)_ = 21.97; *P* < .0001). (C) (1) DA neuron population activity (neurons/track) in response to cannula infusion of amphetamine (cn-AMPH; 10 µM; n = 27, N = 6), caffeine (cn-CAFF; 100 µM; n = 43, N = 5), KW-6002 (cn-KW; 10 µM; n = 51, N = 5), or saline (cn-SAL; n = 39, N = 5) into the NAcc. **P < .01 vs saline; ††*P* < .01 vs caffeine (dashed line); (1-way ANOVA followed by Bonferroni Multiple Comparison Test; F_(3,17)_ = 13.23; *P* < .0001). (2) Firing rate distribution in response to cannula infusion of amphetamine or saline into the NAcc as in C1. Polynomial equations, cn-SAL: y = y = −0.0009x^6^ + 0.0417x^5^ − 0.7813x^4^ + 7.1528x^3^ − 32.278x^2^ + 59.889x − 4E-08; cn-AMPH: y = 0.0085x^5^ − 0.2743x^4^ + 3.266x^3^ − 17.661x^2^ + 39.913x − 0.1082.

A brain circuit involved in the effect of amphetamine on VTA DA neuron activity includes NAcc and VP (Grace, 2010b), and A2A-D2 receptor heteromers of striato-pallidal neurons mediate psychostimulant actions of caffeine (Ferré, 2016). Therefore, the participation of VP in the regulation of DA neuron population activity was investigated by its inactivation through infusion of tetrodotoxin. Thereafter, i.p. injection of either caffeine or KW-6002 failed to affect population activity (Figure 3A1). Amphetamine, in contrast, continued to supress the activity of DA neurons (Figure 3A1; 1-way ANOVA followed by Bonferroni Multiple Comparison Test; F_(4,27)_ = 11.06; *P* < .0001) and to alter the firing frequency distribution (Figure 3A2). Hence, these actions of amphetamine were the same whether VP had been inactivated or not.

From these results, one may conclude that caffeine acts on VP to control VTA DA neurons. To confirm this, drugs were applied directly to VP through cannula infusion. Via this route, both caffeine and KW-6002 decreased population activity, whereas amphetamine failed to do so (Figure 3B; 1-way ANOVA followed by Bonferroni Multiple Comparison Test; F_(4,25)_ = 21.97; *P* < .0001). Thus, responses to caffeine and KW-6002 were similar after i.p. and intrapallidal administration. As amphetamine failed to affect VTA activity when applied to VP, the psychostimulant was infused into the presumed upstream relay NAcc. Thereby, amphetamine diminished population activity of VTA DA neurons (Figure 3C1; 1-way ANOVA followed by Bonferroni Multiple Comparison Test; F_(3,17)_ = 13.23; *P* < .0001) but did not impinge on firing frequency distribution (Figure 3C2). In contrast, infusion of either caffeine or KW-6002 into the NAcc failed to affect population activity (Figure 3C1). Hence, caffeine and amphetamine employed distinct circuits to control VTA DA neurons.

### Repetitive Caffeine Exposure Uncovers A1 Receptor-Mediated Effects on VTA DA Neuron Activity That Differ From Those of Amphetamine

**Figure 4.**
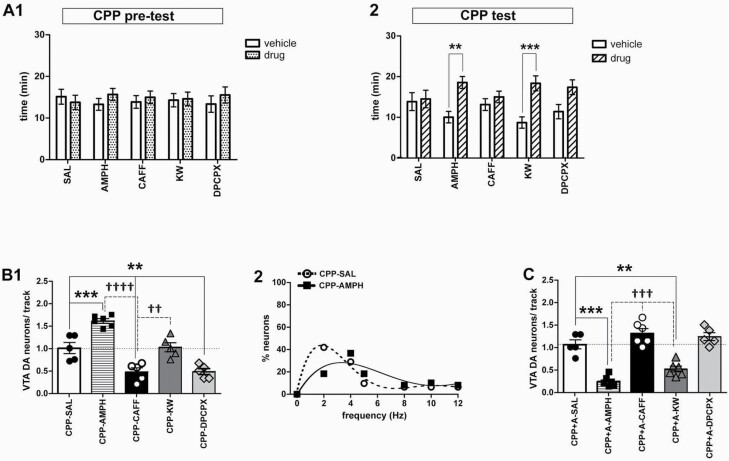
Effects of repeated administration of caffeine and selective adenosine receptor antagonists in conditioned place preference (CPP) and on dopamine neuron population activity: comparison with amphetamine. (A) After 3 conditioning periods in either the drug-paired or vehicle-paired chamber (vehicle), the time spent in either of these 2 during a 30-minute test period was assessed both during pre-test (day = 0; 1) and CPP test (day7; 2); results are shown as total time in minutes spent in each chamber. Drug conditioning was carried out by i.p. injection of (1) amphetamine (AMPH; 1.5 mg/kg; N = 14), (2) caffeine (CAFF; 5 mg/kg; N = 13), (3) KW-6002 (KW; 1.5 mg/kg; N = 14), and (4) DPCPX (4mg/kg; N = 12), respectively. *P < .01 and **P < .001 vs vehicle-chamber, respectively (2-way ANOVA followed by Bonferroni Multiple Comparison test; *P* < .0001). (B) (1) After testing for place preference, DA neuron population (neurons/track) activity was assessed in drug-free animals previously conditioned with amphetamine (CPP-AMPH; n = 63, N = 6), caffeine (CPP-CAFF; n = 21, N = 5), KW-6002 (CPP-KW; n = 38, N = 5), DPCPX (CPP-DPCPX; n = 16, N = 5), and saline (CPP-SAL; n = 36; N = 5), respectively. **P < .01 and ***P < .001 vs saline, respectively; ††*P* < .01 and ††††*P* < .0001 vs caffeine (dashed line) (1-way ANOVA followed by Bonferroni Multiple Comparison test; F_(4,21)_ = 30.78; *P* < .0001). (2) Firing rate distribution after conditioning with either saline (CPP-SAL) or amphetamine (CPP-AMPH); polynomial equations, CPP-SAL: y = 0.0068x^5^ − 0.2664x^4^ + 3.8332x^3^ − 24.097x^2^ + 56.541x − 0.1798; CPP-AMPH: y = −0.009x^4^ + 0.3623x^3^ − 4.6792x^2^ + 20.422x − 1.1885. (C) DA neuron population activity (neurons/track) in response to an additional challenge of drug (drug re-exposure) in mice trained under the CPP protocol. Anesthetized mice received i.p. injections of amphetamine (CPP+A-AMPH; n = 13, N = 6), caffeine (CPP+A-CAFF; n = 52, N = 6), KW-6002 (CPP+A-KW; n = 24, N = 6), DPCPX (CPP+A-DPCPX; n = 47, N = 5), and saline (CPP+A-SAL; n = 38; N = 5), respectively. **P < .01 and ***P < .001 vs saline, respectively; †††*P* < .001 vs caffeine (dashed line) (1-way ANOVA followed by Bonferroni Multiple Comparison test; F_(4,23)_ = 33.56; *P* < .0001).

The NAcc-VP-VTA circuit is part of the brain reward network (Sesack and Grace, 2010). Therefore, adenosine receptor ligands were compared with amphetamine with respect to rewarding properties using a CPP paradigm, and VTA DA neuron population activity was quantified in the same set of mice 30 minutes thereafter. During pre-test, no differences were found within and among the assigned drug treatments (Figure 4A1; 2-way ANOVA followed by Bonferroni Multiple Comparison Test; *P* = .3804). Repeated exposure to amphetamine and KW-6002, but not caffeine or DPCPX, led to drug-related place preference as indicated by an increase in time spent in the drug-paired chamber (Figure 4A2; 2-way ANOVA followed by Bonferroni Multiple Comparison Test; treatment: *P* < .0001, interaction: *P* = .0303). Subsequent VTA recordings revealed that repetitive amphetamine applications led to increased population activity compared with results in mice injected with saline instead (Figure 4B1; 1-way ANOVA followed by Bonferroni Multiple Comparison Test, *P* < .0001). However, repeated amphetamine exposure did not produce a shift in firing frequency distribution (Figure 4B2) as observed after single drug application (Figure 2D1). In mice repetitively exposed to KW-6002, population activity was not different from that in the saline group; in contrast, after repeated administration of either caffeine or DPCPX, population activity was significantly reduced compared with the results obtained after saline injection (Figure 4B1; 1-way ANOVA followed by Bonferroni Multiple Comparison Test; F_(4,21)_ = 30.78 *P* < .0001). Thus, following repeated drug exposure and withdrawal, amphetamine and caffeine elicited opposing responses and the latter appeared to be related to A1 receptor antagonism.

Such persistent changes subsequent to repetitive drug injections provoke the question whether responses to drug re-exposure might be altered as well. To clarify this, mice were trained according to the CPP protocol and received an additional dose of the very same drug prior to the subsequent VTA recordings. In these experiments, amphetamine and KW-6002 reduced numbers of spontaneously active neurons compared with saline. After re-exposure to caffeine, in contrast, values of population activity did not differ from those after saline application, and equivalent results were obtained with the A1 antagonist DPCPX (Figure 4C; 1-way ANOVA followed by Bonferroni Multiple Comparison Test; F_(4,23)_ = 33.56; *P* < .0001). Thus, repeated exposure to caffeine prevented subsequent effects of adenosine A1 receptor antagonism.

## Discussion

All psychostimulants appear to hijack the DA system to elicit motivation, reinforcement, and behavioral activation (Schultz and Dickinson, 2000; Wise, 2004; Nestler, 2005; Koob and Volkow, 2010; Sesack and Grace, 2010). For those that act via the DA transporter, it has been demonstrated that behavioral actions, such as hyperlocomotion, are paralleled by changes in the population activity of VTA DA neurons (Grace, 2000; Grace et al., 2007). The present study confirms these results for amphetamine and reveals a similar correlation for caffeine. Nevertheless, our data uncover conspicuous differences between amphetamine and caffeine with respect to sites of drug actions.

### Caffeine and A2A Receptor Antagonism Orchestrate VTA DA Neuron Population Activity Through Actions on VP

Acute systemic administration of caffeine increased locomotion and reduced VTA DA neuron population activity to an extent similar to that of amphetamine. These effects were most likely mediated by A2A receptors, as they were mimicked by KW-6002, but not DPCPX. It is well known that caffeine exerts biphasic effects on locomotion with enhancement at low to moderate (3–20 mg/kg) and inhibition at higher doses (>30mg/kg). The hyperlocomotion triggered by <20 mg/kg caffeine is lost in A2A knockout mice and also seen with other A1/A2A receptor antagonists, whereas the inhibitory action is mimicked by DPCPX (Griebel et al., 1991; El Yacoubi et al., 2000; Lindskog et al., 2002). Hence, our results regarding locomotion evoked by caffeine or KW-6002 are in full accordance with previous data. The very same doses of these adenosine antagonists reduced VTA DA neuron population activity without changes in average firing frequencies and burst firing pattern. In this respect, caffeine actions are equivalent with those of amphetamine. Nevertheless, there was 1 obvious difference as the firing frequency distribution was altered by amphetamine, as reported previously (Lodge and Grace, 2008), but not by caffeine or KW-6002. This was a first token of different mechanisms being involved in the modulation of VTA DA neuron activity by either adenosine A2A receptor antagonism or DA reuptake reversal.

VP is part of a brain circuit that orchestrates VTA DA neuron population activity (Belujon and Grace, 2017; Grace and Gomes, 2019), and behavioral stimulation by caffeine involves A2A receptors expressed by striato-pallidal neurons (Ferré, 2016). In agreement with this concept, (1) inactivation of VP abolished inhibitory actions of caffeine and KW-6002 on VTA population activity, and (2) infusion of these A2A antagonists directly into VP elicited effects equivalent to those of systemic administration. However, amphetamine effects were not altered by VP inactivation, nor did infusion of amphetamine into VP affect VTA DA neuron population activity. Yet, amphetamine infusion into NAcc did reduce population activity. Hence, analogous actions of amphetamine and caffeine on VTA DA neurons dissociate from each other at the level of VP. Expression of A2A receptors has been detected in the neuropil of VP and localized to GABAergic nerve terminals (Svenningsson et al., 1997; Rosin et al., 1998). Furthermore, these A2A receptors are activated by endogenous adenosine, and its displacement by antagonists leads to a reduction of GABA release (Floran et al., 2005). Accordingly, caffeine can be expected to interfere with inhibitory drive imposed by NAcc medium spiny neurons on GABAergic VP neurons, and the ensuing disinhibition of the latter results in reduced VTA DA neuron population activity. For amphetamine, in contrast, a direct projection from NAcc to VTA DA neurons appeared to be relevant. Data regarding cocaine-induced behavior offer a respective explanation: this DA reuptake blocker was reported to exert effects on locomotion via direct projections of GABAergic NAcc neurons onto VTA DA neurons (Edwards et al., 2017).

### Following Repeated Administration of Caffeine, VTA DA Neuron Activity Is Depressed and Additional Effects of A1 Receptor Antagonism Are Lost

In mice, systemic administration of amphetamine at doses of 1 to 2 mg/kg reliably leads to CPP (Geuzaine et al., 2014; Sukhanov et al., 2016; Wang et al., 2018). Caffeine at 5 mg/kg, in contrast, fails to do so (Muñiz et al., 2017). Equivalent results were obtained in the present CPP experiments. Moreover, CPP was obtained with the A2A antagonist KW-6002, but not with the A1 antagonist DPCPX, and this is in line with previous data on DPCPX and a selective A2A antagonist other than KW-6002 (Hsu et al., 2009).

Subsequent to CPP experiments, mice that had been injected with amphetamine displayed enhanced VTA DA neuron population activity, whereas those exposed to caffeine had reduced population activity. The latter results were mimicked by DPCPX but not KW-6002. Our results regarding amphetamine are in line with previous results demonstrating that chronic exposure to amphetamine followed by withdrawal elicited behavioral sensitization and increased DA neuron population activity (Lodge and Grace, 2008). For caffeine, the repeated administration led to a switch from the restriction of VTA DA neuron population activity via most likely A2A receptors, as observed after single-dose application, to a sustained depression that was mediated by A1 rather than A2A receptors. In behavioral experiments, chronic caffeine intake reduces locomotor activity of mice and attenuates inhibitory effects of adenosine receptor activation thereon (Nikodijević et al., 1993). Possibly, the continuous inhibitory effect on both VTA DA neuron population activity and locomotor behavior following repeated caffeine exposure can be related to changes in plasma adenosine concentrations (Conlay et al., 1997) and/or to overexpression of A1, but not A2A, adenosine receptors in the striatum as observed in previous studies (Shi et al., 1994; Karcz-Kubicha et al., 2003). In this context, it should be mentioned that actions of amphetamine on NAcc neurons may involve activation of presynaptic A1 receptors by adenosine derived from astrocytes (Corkrum et al., 2020).

In animal models of depression, such as chronic mild stress or learned helplessness, population activity of VTA DA neurons has been found to be reduced and to be enhanced in response to antidepressant drugs such as ketamine (Moore et al., 2001; Belujon and Grace, 2014). Hence, the reduced activity observed after repetitive caffeine administration can be viewed as a correlate of depressed mood, one of the symptoms of caffeine withdrawal in humans (Juliano and Griffiths, 2004).

When mice were re-exposed to the very same drugs as used during CPP experiments, amphetamine and KW-6002 were able to reduce VTA DA neuron population activity compared with saline, but caffeine and DPCPX failed to do so. The latter result is in line with findings showing that chronic caffeine treatment prevents subsequent modulation of DA release in the NAcc by A1, but not A2A antagonists (Quarta et al., 2004), and leads to tolerance towards behavioral effects of A1, but not A2A blockers (Karcz-Kubicha et al., 2003). Moreover, chronic cocaine exposure has been found to attenuate inhibition of glutamate release by endogenous adenosine acting on presynaptic A1 receptor (Manzoni et al., 1998). This apparent lack of effect of A1 receptor antagonism after habituation to caffeine can be viewed as a correlate of caffeine tolerance in humans (Juliano and Griffiths, 2004).

Reapplication of amphetamine and KW-6002 after preceding repetitive exposure to the very same drug, in contrast, resulted in reduced values of VTA DA neuron population activity. Hence, there was no evidence for a development of tolerance to these agents. Thus, regarding the orchestration of VTA DA neuron population activity, the transition from single acute to repeat caffeine exposure is accompanied by a switch from A2A to A1 antagonism as apparently predominating mechanism. Possibly, this switch is fundamental with respect to differences in the chronic psychostimulant actions of caffeine and amphetamine, respectively.

Taken together, behavioral effects of caffeine and amphetamine as well as actions on VTA DA neurons are comparable after single dosing but differ after repeated drug exposure, as sensitization of the DA system is seen with amphetamine, but not with caffeine.

## Notes

S.B. received support from the interuniversity cluster project “Novel scaffolds for improved antiepileptic drugs” financed by the University of Vienna and the Medical University of Vienna.
